# NGS in Lung, Breast, and Unknown Primary Cancer in Colombia: A Multidisciplinary Consensus on Challenges and Opportunities

**DOI:** 10.1200/GO.21.00046

**Published:** 2021-06-29

**Authors:** William Armando Mantilla, María Carolina Sanabria-Salas, Ana Margarita Baldion, Luz F. Sua, Diego Mauricio Gonzalez, Mauricio Lema

**Affiliations:** ^1^ Fundacion Cardioinfantil, Universidad del Rosario, Bogotá, Colombia; ^2^ Instituto Nacional de Cancerología, Bogotá, Colombia; ^3^ Head of the Pathology Section, Department of Pathology and Laboratory Medicine, Hospital Universitario Fundacion Santa Fe de Bogota, Bogotá, Colombia; ^4^ Department of Pathology and Laboratory Medicine, Fundación Valle del Lili, and Faculty of Health Sciences, Universidad ICESI, Cali, Colombia; ^5^ Instituto de Cancerología Las Americas-AUNA, Universidad de Antioquia, Antioquia, Colombia; ^6^ Clínica de Oncología Astorga, Medellín, Colombia

## Abstract

Given the benefits and likely future applications, there is an urgent need to expand the use of next-generation sequencing (NGS) in breast, lung, and unknown primary cancers in Colombia. The objective of this review is to address the barriers limiting access to the use of NGS in Colombia, specifically for patients with breast, lung, and unknown primary cancers in the public health care system. A selected Panel of Colombian experts in NGS were provided with a series of relevant questions to address in a multiday conference. Each narrative was discussed and edited by the Panel through numerous drafts and rounds of discussion until consensus was achieved. There are limitations to the widespread adoption of innovative technology inherent to the Colombian health care system. Barriers identified to implementing NGS in Colombia include availability, accessibility, and affordability; limited infrastructure; training and awareness of health personnel; quality-control procedures; and collection of local data. Stakeholders must align to adapt the implementation of NGS to the constraints of resource-limited environments. Diagnostic algorithms were developed to guide molecular testing for lung, breast, and unknown primary cancers. Recommendations on overcoming the barriers to the widespread adoption of NGS include country-specific molecular testing guidelines, creating a national genetic registry, improving infrastructure, and creating health policy that favors the adoption of innovative technology.

## INTRODUCTION

A new generation of sequencing technologies represents an immense opportunity for precision medicine (PM) in patients with cancer: next-generation sequencing (NGS).^1^ Understanding the molecular and genetic properties of tumors has wide applications in oncology research and clinical practice, with resulting diagnosis and treatment advantages.

**CONTEXT**

**Key Objective**
This article addresses the question of how integrated genomic research projects, accessible next-generation sequencing (NGS) platforms, thoughtful NGS-associated educational programs, and inclusive health policies regarding NGS can be implemented to increase NGS access in Colombia.
**Knowledge Generated**
NGS provides diagnostic, therapeutic, and prognostic indications, leading to improved patient outcomes in those with lung, breast, or unknown primary cancer. Addressing a complex regulatory environment, economic constraints, the intricate nature of the bioinformatics infrastructure, and highly trained personnel who can interpret NGS techniques is necessary to improve access to NGS. Despite these challenges, NGS implementation provides the opportunity for wide-ranging solutions in cancer care through the development of molecular testing guidelines, enhanced data collection, state-of-the-art technology, molecular tumor boards, necessary government regulations, and increased patient access.
**Relevance**
The findings described in this article offer recommendations for advancing NGS capabilities that improve cancer diagnosis, pinpoint potential management options, and innovate cancer treatment in Colombia.

NGS can be used to predict disease risk, characterize molecular alterations, and define possible therapies on the basis of genetic alterations.^2^ In line with global trends, the Latin American (LA) market is projected to grow at a compound annual rate of 14.3% and reach US dollars 714.8 million by 2027.^3^ Colombia is one of the largest LA countries with more than 50 million inhabitants, and more than 100,000 new cancer cases diagnosed annually.^4^ Considering the growing and aging population, NGS is becoming increasingly necessary. Thus, obstacles within the Colombian health care system that challenge implementation must be recognized.

## METHODS

To address these issues, the Americas Health Foundation (AHF) conducted a literature review to identify scientists and clinicians from Colombia who have published on NGS. Using PubMed and Embase, AHF identified clinicians and scientists with an academic or hospital affiliation and who had published in molecular oncology since 2015. Augmenting this search, AHF contacted several individuals in Colombia to tailor a list of Panel members who could create nation-specific recommendations. As a result of this effort, AHF convened a six-member Panel of clinical and scientific experts from Colombia, representing the disciplines of precision medicine (PM), oncology, pathology, and genetics. Great attention was paid to ensure a diverse Panel representing various disciplines related to NGS.

### Search Strategy and Selection Criteria

Papers useful for the consensus discussion and the references cited in this article were identified through searches of PubMed and Embase with the search terms NGS, molecular testing, targeted therapy, and next-generation sequencing from 2013 until 2020. Additional articles were identified through bibliographies of the papers identified in the search and from the authors' own files. Particular attention was paid to papers that reviewed or summarized the topic in question or were related to activities in Latin America, especially Colombia. The final reference list was generated on the basis of the relevance to the broad scope of this consensus document.

AHF staff developed specific questions for the Panel to address, which addressed salient issues. Subsequently, each individual Panel member wrote a response to each question, which the entire group edited through numerous drafts and rounds of discussion. During November 3-5, 2020, each question was discussed at length, and an outline for the answer to each question was established. After the meeting, the Panel reviewed the document to again acknowledge it was in full agreement.

## COLOMBIAN HEALTH SYSTEM AND NGS

To understand the current limitations for molecular technology implementation, Colombia's public and private health care systems must be outlined. The General Social Security Health System (SGSS) is the public system covering 90% of the population. It includes two regimens: contributory and subsidized, both of which are funded through general taxation and payroll contributions. The contributory regimen covers salaried and independent workers and those who are pensioned (approximately 47%), the subsidized regime covers anyone who cannot pay (approximately 48%), and the remaining are considered an exceptions regimen (approximately 4%). The 5% that lack coverage are salaried, recently unemployed, or independent workers who are unable to meet the compulsory monthly payment but do not qualify for the subsidized regimen. Enrollment in the public system is compulsory, and the insurers are health promotion agencies (EPS). Health care is provided by institutional health service providers (IPS) that may or may not be part of the EPS.^5,6^ Nonetheless, specialized medical care is not always covered.^7^ The Basic Healthcare Plan (PBS), established by the Committee of Health Regulations, is a list of services and interventions covered by the EPS. If an intervention is excluded by this list, additional physician justification or legal action is required.

Colombia's oncology practice is highly restrictive, in part because only technologies with regulatory approval can be used with a reasonable coverage expectation. Although patients with cancer can expect full health care coverage for care outlined within national clinical practice guidelines (CPG), these include limited consideration of molecular-guided therapy.^8^ Innovative strategies, such as targeted therapy, are subjected to a long, expensive process to achieve government approval. If the technology is available but not accessible, off-label use authorization must be requested, which can be still rejected by the EPS but legally and financially bind the prescribing IPS under the law of maximum budgets. Decisions issued by tumor boards and treating physicians must be recognized by national entities and EPS, respecting medical autonomy and optimizing public spending.^9^ Aspects of Colombian health care that create barriers to innovative health care inclusion and access are discussed below.

### Single Procedure Classification in Health

The single procedure classification in health, an official code list of medical procedures and services, was created to standardize data and homogenize medical procedure language used between all SGSS actors. This classification includes few, nonspecific codes for molecular tests, creating a significant access barrier. Updating the list to include codes and descriptions required for molecular testing is necessary. These codes should be congruent with national test and treatment availability.^10^

### Limited Molecular Biology Laboratories

Very few public or private Colombian laboratories offer standardized NGS in-house tests, partly because such complex technology (ie, technical expertise, bioinformatics and computing infrastructure, and data interpretation) is challenging to implement. To perform molecular biology testing, laboratories must meet the minimum licensing standard for operation (*habilitación*). The approval specifications for molecular biology laboratories are included within the specifications for clinical laboratories; however, not every IPS with clinical laboratory *habilitación* has the resources to establish a molecular biology section.^11^ In these cases, laboratories must send samples abroad, which is not always possible.

### National CPGs

Currently, standardized guidelines specific to molecular testing, let alone NGS, do not exist in Colombia, creating variability in cancer care access. Currently, CPGs only address molecular diagnosis and treatment for lung cancer (LC) and breast cancer (BC).

Although the latest BC guideline includes algorithms for hereditary breast and ovarian cancer (HBOC) on the basis of England's National Institute for Health and Care Excellence recommendations, these are considered restrictive by most physicians, who regularly adhere to National Comprehensive Cancer Network guidelines.^12^ To standardize care, protocols regarding HBOC identification and management must be established in Colombia because testing decisions are currently left to physician criteria.^13^ PBS includes germline testing for high-risk patients in hereditary cancer syndromes; although somatic testing is available, additional regulatory hurdles exist that restrict access and delay treatment. For LC, molecular-guided therapy is less standardized. The latest national LC guideline from 2014 only mentions epidermal growth factor receptor (*EGFR*) testing and guided therapy for lung adenocarcinoma as optional, without mention of programmed death ligand 1 testing or specific therapy.^14^ A Colombian consensus on advanced LC addressed *PDL1*, *EGFR*, *ALK*, and *ROS1*.^15^ Molecular testing for cancer of unknown primary (CUP) is not covered in any national guideline.

### Data Collection

NGS has unlocked PM opportunities. To implement novel molecular-guided therapies, standardized guidelines that establish specific molecular testing indications, on the basis of local prevalence, test availability, and international recommendations, must be created. Therefore, local data collection and generation are crucial by leveraging research collaborations that explore local epidemiology and genetic variations.

### Molecular Tumor Boards

Molecular tumor boards are key to guide clinical oncology decision making^16^ and may be beneficial for NGS application. This approach allows for patient-specific therapeutic strategies by identifying target alterations through NGS.^16^ Additionally, tumor boards with comprehensive NGS training on clinical and ethical issues would allow for a better approach to NGS application, data interpretation, clinical integration, and ethical implications.^16,17^ Despite challenges that PM faces within Colombia, three cancers have relevant NGS applicability: LC, BC, and CUP. The Panel identified specific cases when NGS use is efficient and outlined recommended algorithms for molecular testing in these cases.

## LC IN COLOMBIA

### LC Epidemiology

LC incidence and mortality have risen dramatically in Latin America,^18,19^ which could reflect inadequate tobacco control policies.^19^ Colombia remains consistent, with the age-standardized incidence and prevalence rates of LC of 10.1 and 10.8 per 100,000 inhabitants respectively. Conversely, the LC mortality rate in Colombia is 9.0 per 100,000 inhabitants, which is comparatively high. These figures are discrepant with local data on which payer decisions are based (high-cost account), which reports a 6.4 prevalence and a 2.3 mortality.^20^ In Colombia, LC prognosis is poor, with an expected 5-year survival rate of 8.7%, and approximately 85% of LCs are non–small-cell lung cancer (NSCLC).^21^

### Management Decision Making

Surgical treatment is recommended for stage I and II LC for those eligible and in patients with stage IIIA LC who respond to preoperative chemotherapy or radiotherapy. Metastatic NSCLC treatment is based on histology, performance status, CNS involvement, PDL1 expression, and actionable targets, including recurrent genomic alterations. Targeted therapy is the preferred treatment in patients with actionable mutations, both at diagnosis and at progression, such as *EGFR*, *ALK*, and *ROS1*, which NGS detects.^22^ As more agents are approved, more testing will be necessary; thus, tissue exhaustion, an already-existing problem, will become a more pressing issue. Parallel sequencing of multiple genes, such as NGS, may provide a solution. Routine analysis of gene alterations is currently limited to advanced and metastatic disease in clinical practice; however, it may be useful in early disease.^23,24^

### Nonsquamous NSCLC

*EGFR* mutations are frequent in the Colombian population (24.7%), which may be because of a large indigenous ancestry (approximately 29%).^25,26^ *ALK* fusions, another predictive biomarker, occur in approximately 3% of nonsquamous NSCLC and must be identified because ALK inhibitors are highly effective.^27^ Immunohistochemistry (IHC), fluorescent in situ hybridization (FISH), and RNA sequencing detect *ALK* positivity. Other recurrent actionable alterations include *MET*, *BRAF*^*V600E*^, *ROS1*, *NTRK*, and *RET* (Table 1). For each, specific agents exist with high, often long-lasting, response rates.

**TABLE 1:**
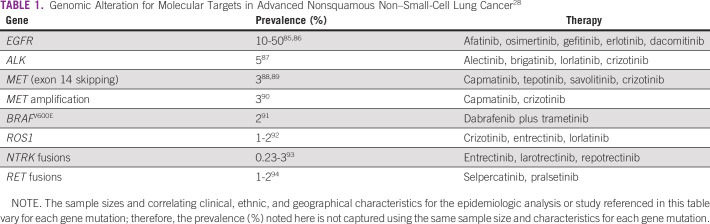
Genomic Alteration for Molecular Targets in Advanced Nonsquamous Non–Small-Cell Lung Cancer^28^

On the basis of the high frequency of clinically significant and actionable genomic alterations, the European Society for Molecular Oncology (ESMO) recommends NGS for advanced nonsquamous NSCLC.^28^ Adhering to this in Colombia is difficult because few agents for these alterations are approved. As such, only *EGFR* and *ALK* mutation testing are routinely performed. From a research perspective, the absence of comprehensive genomic characterization is a barrier to including Colombian patients in molecular-driven trials.^29^

Recently, the ADAURA trial changed the therapeutic landscape of nonmetastatic disease, integrating molecular markers in treatment selection^23^ with improvements in disease-free survival. On the basis of this, *EGFR* testing should be considered for adjuvant osimertinib therapy for lung adenocarcinoma.^23^ Currently, osimertinib is not approved for the adjuvant setting in Colombia. *EGFR* testing in early nonsquamous NSCLC is contingent upon the approval of osimertinib.

Patients with stage IIIC LC have a 5-year survival rate of 13%.^30^ Standard of care in this subgroup is chemoradiotherapy. For patients ineligible for chemoradiotherapy, early actionable target identification could allow targeted therapy. In metastatic disease, a complete molecular profile is mandatory since there are multiple actionable therapeutic targets, molecular profiles, and PDL1-based therapies. Consequently, patients with a therapeutic target who receive targeted therapy have a better prognosis than those who do not. In one study, the median overall survival was 2.4 years in patients with actionable mutations not initially treated with a targeted agent, compared with a median overall survival of 3.5 years in those who were.^31^

**FIG 1:**
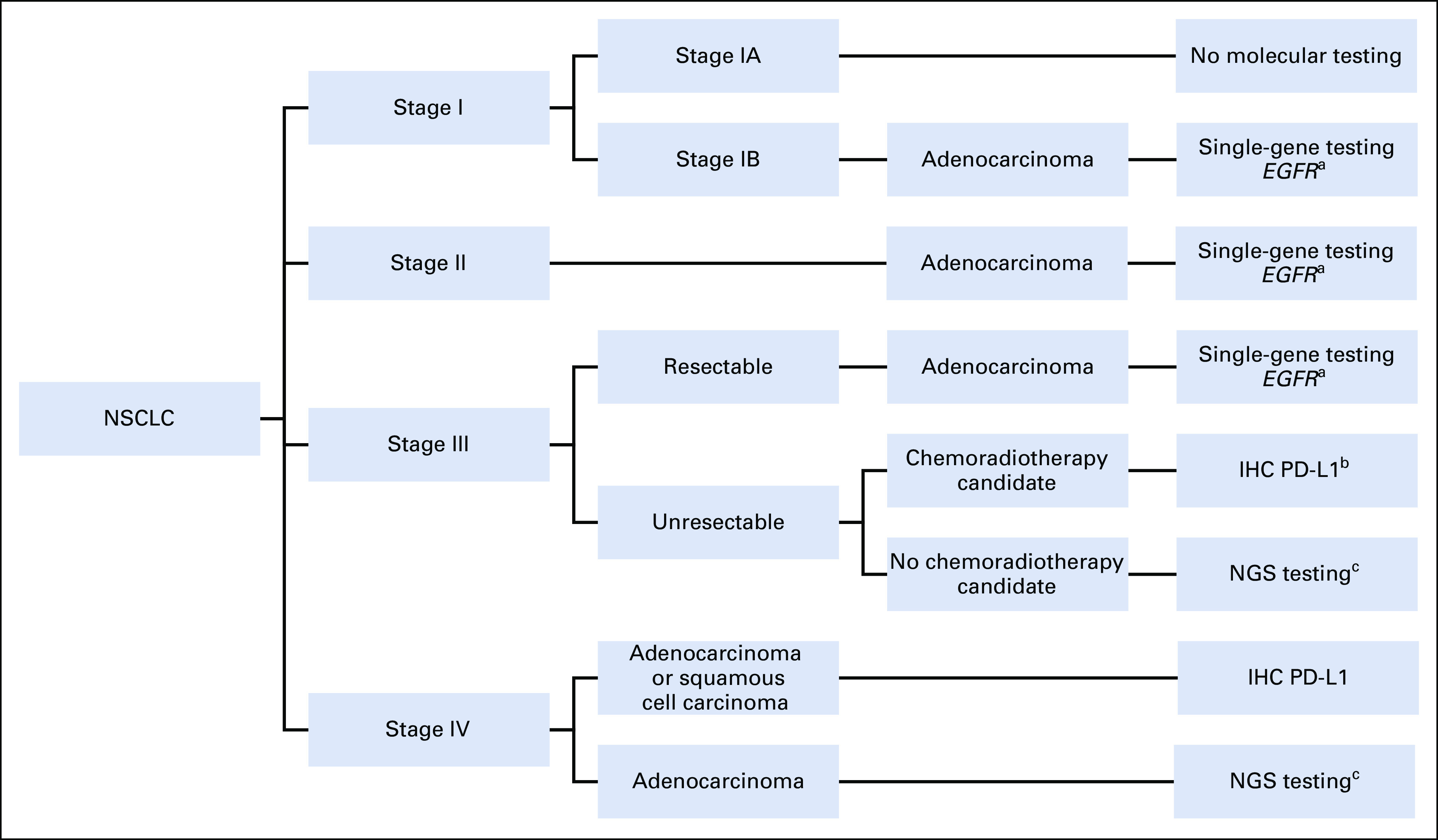
Algorithm for integrating molecular testing according to LC stage. ^a^Currently, osimertinib is not approved for adjuvant treatment in the country. ^b^Durvalumab treatment should be considered on the basis of PD-L1 expression and at least stable disease after chemoradiotherapy treatment,^95^ ^c^NGS testing in stage III and IV NSCLC could be considered in nonadenocarcinoma tumors including young never smoker patients with squamous cell carcinoma, or in adenosquamous cell carcinoma. *EGFR*, epidermal growth factor receptor; IHC, immunohistochemistry; LC, lung cancer; NGS, next-generation sequencing; NSCLC, non–small-cell lung cancer; PD-L1, programmed death ligand 1.

Initially, sequential tests were performed to determine *EGFR* mutations, followed by *ALK* analysis by IHC, FISH, or sequencing. However, given the number of identifiable markers, performing an NGS molecular panel could improve alteration identification and avoid tissue exhaustion.^15^ NGS panels have shown a higher performance compared with hotspotting or single-gene testing because of the number of alterations found.^32^ Recently, ESMO published NGS recommendations, suggesting that advanced or metastatic lung adenocarcinoma should be tested with an NGS molecular panel.^28^ Figure 1 shows a flowchart to guide molecular testing in patients with NSCLC.

## BC IN COLOMBIA

### BC Epidemiology

BC, the leading mortality cause in Colombian women, is a highly heterogeneous disease with different molecular patterns, prognoses, and therapeutic responses.^33-36^ In 2018, approximately 13,400 new BC cases were diagnosed in Colombia (44.1 per 100,000 inhabitants), representing 13.1% of total cancer diagnoses.^37^ These figures reflect deficiencies in screening, diagnosis, and treatment.^38^

According to the global histologic and IHC classification, BC is divided into four clinically relevant subtypes: luminal A, luminal B, human epidermal growth factor receptor 2 (HER2)–enriched, and triple-negative breast cancer. In Colombia, luminal B is the most prevalent (37.2%-50%) and triple-negative breast cancer the second (21%).^33,39,40^ BC subtypes are key prognosis determinants and predict treatment response.^41^ The standard of care is IHC for estrogen receptor, progesterone receptor, Ki 67, and HER2.^33,39-41^ NGS use and gene profiling in BC focused on identifying HBOC and specific early BC subtypes (ie, claudin-low androgen receptor, enriched mesenchymal-like, basal-like luminal HER2-enriched).

Disadvantages in health service access result in more advanced cancers and greater treatment delays.^42^ The Instituto Nacional de Cancerología (INC), the leading public cancer center in Colombia, reported around 65% of patients with BC in the public system were diagnosed at advanced stages, compared with 40% in the private system.^43^ Disparities between time to first consultation and treatment initiation were found between the systems.^43^

### Hereditary BC Syndrome

Global HBOC prevalence ranges from 5% to 10%,^44^ but variable data exist for Colombia, with reported *BRCA1* and *BRCA2* mutation frequencies from 1.2% to 24.5%.^45-48^ Two studies used NGS multigene panels,^47,48^ which identified other genes linked to hereditary BC syndromes.^48^ Nevertheless, 60%-80% of HBOC cases are related to *BRCA1* and *BRCA2* mutations.^47,48^ In Colombia, *BRCA* mutation testing is covered by the health care system, but germline testing turnaround takes several months, often reducing the utility for clinical decision making.^49^ Identifying high-risk individuals and families through genetic counseling and germline testing and implementing surveillance and screening, chemoprevention, and risk-reducing surgeries are strategies that will reduce BC's economic burden in the medium to long term.^50-52^

These steps should be standard of care in clinical practice, ideally supported by a multidisciplinary team for a comprehensive patient and family approach, identified through cascade testing.^53,54^ Through this process, true negatives must be identified because their cancer risk is considered comparable with the general population. Elucidating the variants of uncertain significance found in the Colombian population (35%-41%) is crucial.^47,48^ Public databases contain < 1% of LA genetic data^55^; therefore, increased genetic analyses in the Colombian population will promote reclassification of variants of uncertain significance specific to the Colombian population.^56^

### Gene Expression Profiling and Comprehensive Molecular Profiling

A 21-gene assay expression profile (OncoTypeDx) can identify patients unlikely to benefit from adjuvant cytotoxic chemotherapy in stage I, invasive, luminal type BC (estrogen receptor–positive/progesterone receptor–positive/HER2-negative).^57^ The TAILORx phase III trial proved that chemotherapy can be safely avoided in low- and intermediate-risk patients. This result is paramount because approximately 80% of postmenopausal and 50% of premenopausal women have low or intermediate scores.^57,58^ Less robust clinical trials with other assays have shown consistent, albeit less dramatic, results. National CPGs recommend requesting genomic expression profiles in patients with early BC with T1 and T2, negative nodes, positive hormone receptors, and HER2-negative.

**FIG 2:**
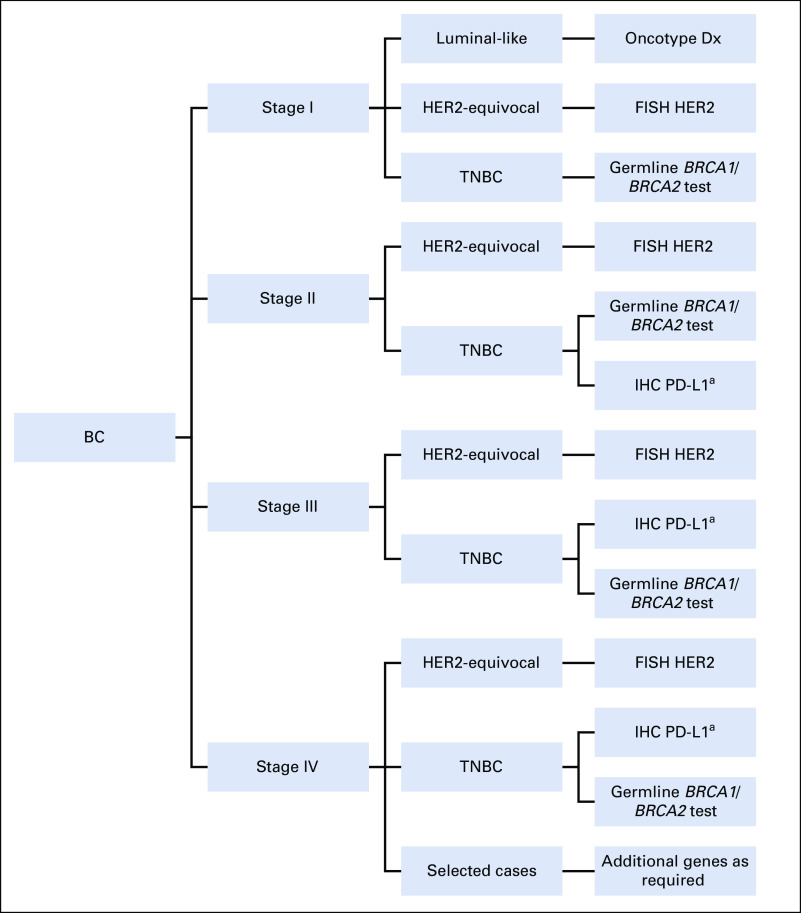
Algorithm for integrating molecular testing according to BC stage. ^a^Only if neoadjuvant therapy is being considered. BC, breast cancer; Dx, diagnosis; FISH, fluorescent in situ hybridization; HER2, human epidermal growth factor receptor 2; IHC, immunohistochemistry; PD-L1, programmed death ligand 1; TNBC, triple-negative breast cancer.

Germline testing for *BRCA1* and *BRCA2* mutations may have therapeutic implications since evidence supports poly (ADP-ribose) polymerase inhibitor response in patients with *BRCA1*- and *BRCA2*-mutated and metastatic BC.^59,60^ Olaparib and talazoparib, poly (ADP-ribose) polymerase inhibitors, improve progression-free survival in germline *BRCA1*- and *BRCA2*-mutated metastatic BC. The latter improves pathologic complete response in early-stage BC. *PIK3CA* mutations, identifiable through polymerase chain reaction and NGS, occur in up to 40% of BC. A clinical trial showed that alpelisib, a PIK3CA inhibitor, plus fulvestrant improved outcomes in *PIK3CA*-mutated luminal metastatic BC.^61^ Alpelisib is not available in Colombia; therefore, *PIK3CA* testing is contingent on therapy approval. *ERBB2* (HER2) amplifications are predictive of clinical benefit with anti-HER2 therapies, such as neratinib.^62,63^ *ESR1* mutations arise in patients treated with aromatase inhibitors and modify endocrine therapy response.^64^ Microsatellite instability (outside of Lynch syndrome) and *AKT* and *NTRK* fusions are rare in BC. Without treatment recommendations on the latter, NGS testing is not routinely recommended in BC, and a single-gene testing approach is valid.^28^ Figure 2 shows an algorithm to guide molecular testing in patients with BC.

### Management Decision Making

BC treatment consists of surgery, radiotherapy, and systemic therapy. Chemotherapy treatment is based on clinical BC stage and phenotype, which are used as surrogates for relapse risk after initial management in nonmetastatic BC.^65^ However, available molecular profiling tools can determine adjuvant chemotherapy indications. For metastatic BC, systemic therapies are a treatment cornerstone. Multiple chemotherapeutic regimens, endocrine therapies, and some target agents are available for BC.

## CUP IN COLOMBIA

### CUP Epidemiology

CUP is a metastatic disease without an identified primary tumor at diagnosis, despite following a standardized diagnostic approach.^66,67^ Globally, CUP epidemiologic data are scarce. In Colombia, approximately 2,000 new CUP cases are diagnosed annually, with decreasing mortality, in line with global trends.^68,69^ Adenocarcinoma and undifferentiated tumor are the most common CUP in Colombia (37.5% and 27.5%, respectively).^70^

### CUP Diagnosis

Approximately 80% of patients with CUP have unfavorable outcomes, raising diagnostic and treatment challenges.^71,72^ Patients presenting CUP should undergo a thorough clinical and paraclinical examination, and, most importantly, a histopathologic, molecular, and IHC analysis. If no primary site is established after this workup, it will remain unidentified in 70%-90% of cases.^66,73^ Colombia does not have a CUP guideline, resulting in unstandardized care.

Effective CUP diagnosis is influenced by high-quality histopathology, standardized diagnostic algorithms, and direct communication between pathologists and oncologists. Primary goals of IHC analysis are to determine cell lineage (carcinoma, melanoma, lymphoma, or sarcoma), differentiate tumor subtypes (adenocarcinoma, hepatocellular, renal, thyroid, germ cell, squamous, or neuroendocrine), and determine adenocarcinoma primary site.^67^

### Decision Making in Management

CUP treatment is based on the recognition of favorable prognosis subgroups using clinical and pathologic criteria.^67^ Favorable prognosis of CUP treatment can be homologated to several treatment algorithms of corresponding cancers of known primary site. The remaining patients with CUP are deemed unfavorable prognosis, and the majority are treated with broad-spectrum combination chemotherapy, with a median overall survival of 11 months.

### CUP Molecular Studies

**FIG 3:**
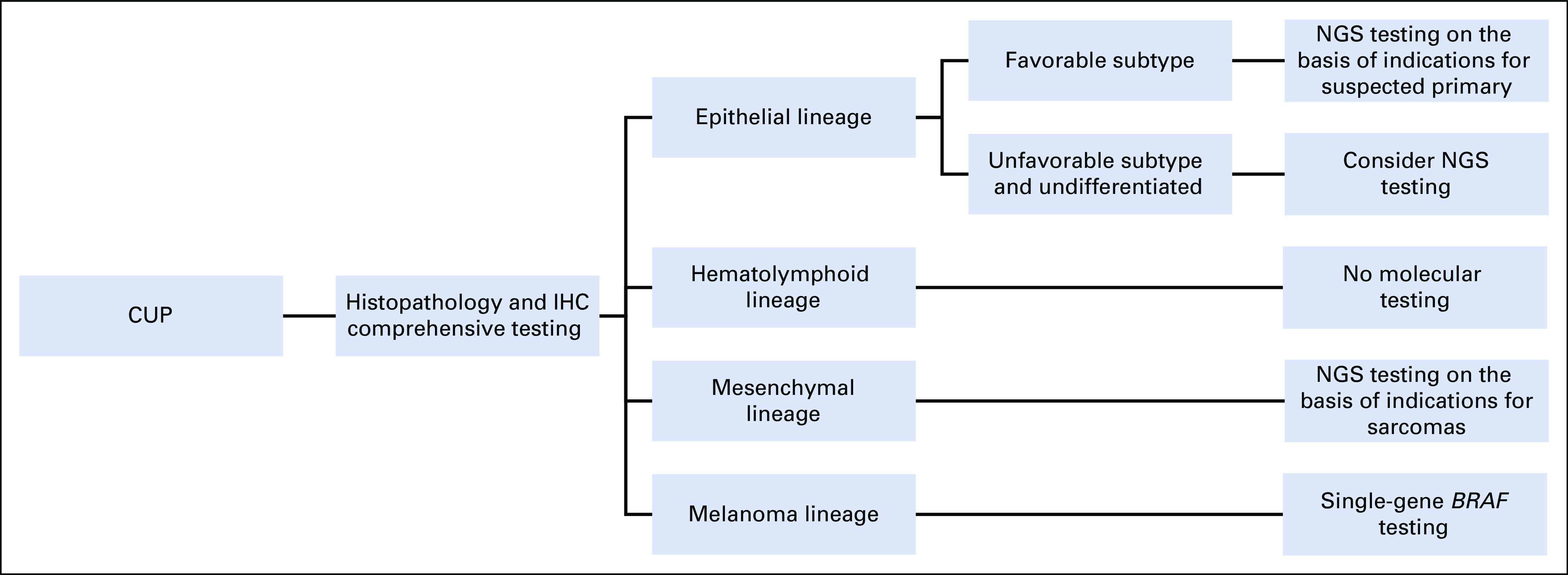
Algorithm for integrating molecular testing according to CUP cell lineage. CUP, cancer of unknown primary; IHC, immunohistochemistry; NGS, next-generation sequencing.

The two CUP testing approaches are molecular profiling and NGS. Some trials based on gene expression profiling have shown an impact on CUP patient survival, specifically when actionable alterations can be identified. Increased CUP survival was observed with molecular profile testing, with treatment on the basis of gene expression profiles,^74^ which are currently not available in Colombia. The second approach is NGS-based. Up to 52% of CUP have clinically meaningful tumor-agnostic alterations, including mutations in *PIK3CA*, *ERBB2*, *BRAF*, and *NTRK*, and molecular signatures such as microsatellite instability–high and tumor mutational burden–high.^75^ Actionable mutation-driven treatment is currently being evaluated by the CUPISCO trial. However, even when actionable mutations are identified, therapies related to these alterations are usually not approved for CUP; thus, strategies for granting agnostic drug access are needed. Molecular testing, recommended by ESMO, is crucial and should be performed in all patients with undifferentiated carcinoma after an initial clinical and histopathologic approach.^28^ Figure 3 shows a proposal for the CUP diagnostic approach.

## NGS IMPLEMENTATION BARRIERS

Despite the many advantages of using NGS in these cancers, factors inherent to NGS and the Colombian health care system create significant implementation obstacles. A complex regulatory environment, economic constraints, the intricate nature of the bioinformatics infrastructure, and trained personnel required create challenges that must be addressed. The principal barriers to NGS adoption in Colombia are discussed below.

### Availability, Accessibility, and Affordability

As outlined previously for each cancer, some targeted therapies are either not available or not accessible in Colombia because of the lack of coverage or approval in PBS, on which NGS approval is contingent. Additionally, vast access inequities exist in available therapies between the public and private health care systems. Tests that identify therapeutic targets often are financed by pharmaceutical companies, which favors access. The price of NGS has decreased in Colombia; nonetheless, the cost remains four to five times higher than in other countries because of taxes, analysis costs, shipping costs, and required infrastructure.^76^

### Infrastructure

NGS requires a robust infrastructure of human, technologic, financial, and bioinformatic resources that most institutions in Colombia do not have. An oncology specialist shortage leads to overburdening and treatment delays and hinders tumor board implementation, which are generally unavailable. Pathology laboratories must receive appropriate investments for technologic infrastructure necessary for molecular testing. Although Colombia recognizes cancer as a priority,^77,78^ NGS must receive appropriate resource allocation for development.^79^ A constitutional court ruling identified the need for spending on cancer prevention and early detection through NGS.^80^

### Training and Awareness

NGS requires highly trained personnel in bioinformatics, genetic counseling, and basic biomedical sciences in oncology. Several training programs were created, but more programs are needed for medical personnel to enter these fields.^81^ An informed medical community and a highly coordinated environment between the different disciplines in molecular testing are required. Physicians handling NGS results must be trained to manage the ethical implications of incidental findings. All stakeholders involved in the decision-making process must be aware of the benefits of routine NGS use on outcomes, cost-effectivity, and overall health expenditure.

### Quality-Control Procedures

Adequate quality-control standards that regulate sample preparation, data interpretation, and results do not currently exist in Colombia. Minimum quality measures (eg, coverage, depth, variant allele frequency, and tumor representation proportion) should be included in genetic testing reports.^82-84^

### Research and Data

Studies that collect data to characterize the Colombian population, enabling accurate treatment decisions and sound public policy, are lacking. Creating national genetic data registries would establish the true significance of country-specific cancer-related variants. A collaborative environment that promotes NGS research in outcomes, impact, and cost-effectiveness in Colombia is needed.

## CONCLUSIONS AND RECOMMENDATIONS

NGS implementation in developing countries must be adapted to the constraints of resource-limited environments. In addition to indicating therapies, NGS can provide prognostic information, which undeniably benefit patients with LC, BC, or CUP. As more international guidelines endorse NGS, stakeholders must align to incorporate this technology in health systems to improve cancer care. NGS implementation highlights several preexisting problems within the Colombian health care system and provides the opportunity for wide-ranging oncology solutions. Solutions for streamlining regulatory processes, cancer care standardization, and homogeneous decision making are outlined below:

1. National clinical practice guidelines
  a. Molecular testing guidelines must be developed on the basis of local data by a multidisciplinary team for clinical PM application, including recommendations for molecular tests such as NGS, gene expression profile, FISH, chromogenic in-situ hybridization, and dual in-situ hybridization.
  b. Medical societies, IPS, EPS, and government must adapt existing BC and LC guidelines to include genetic and molecular diagnosis and treatment pathways alongside Colombian health system stakeholders.
  c. The government agencies, Institute of Technological Evaluation in Health, and medical societies should collaborate to align government access regulations and national guidelines on the basis of impartial knowledge, high-quality clinical trials, and cost-effectiveness.
  d. Government should ensure NGS is included in PBS and physicians should order NGS in the following scenarios:
    i. Provide NGS for all advanced nonsquamous NSCLC to identify actionable targets for the therapies available, including *EGFR*, *ALK*, *ROS1*, *BRAF*, *MET*, and *HER2*.
    ii. Ensure access to genetic testing and genetic counseling for HBOC.
    iii. Perform NGS for all unfavorable prognosis CUP because actionable targets can be potentially life-altering.
    iv. Routinely perform NGS when multiple genes must be studied for somatic and germline mutations as it is the most efficient approach to perform parallel testing of multiple genes.
2. Data generation and collection
  a. Sistema Nacional de Ciencia Tecnología e Innovación alongside academic institutions and medicals societies should do the following:
    i. Generate local data through a country-specific genetic registry and leveraging research collaborations that provide local epidemiology and genetic variations
    ii. Prioritize research on PM's impact
    iii. Conduct pharmacoeconomic analyses to provide evidence for PM inclusion
3. Human and technologic resources
  a. Stakeholders must facilitate the development of high-quality laboratories that can perform state-of-the-art molecular testing by the following ways:
    i. Investing in pathology laboratories specialized in molecular testing to allow for the required infrastructure (government and health institutions)
    ii. Adapting quality standards that regulate molecular biology testing at all levels and standardize NGS reports (government, health institutions, and medical societies)
    iii. Addressing the specialist shortage by increasing training programs for bioinformatics, molecular biology, and genetic counseling (academic institutions and MinCiencias)
4. Molecular tumor boards
  a. Health institutions must create molecular tumor boards that include oncologists (clinical and surgical), pathologists, geneticists, bioinformatic experts, bioethicists, and administrative personnel to ensure a comprehensive approach.
  b. Health institutions must address access inequalities by developing a network of molecular tumor boards that can meet the population's PM needs.
5. Patient access
  a. Government must create health policies that allow opportune access to innovative oncology technology and allocate appropriate funding.
  b. National regulatory entities and insurers must recognize and respect medical autonomy.
  c. The Ministry of Health must address data discrepancies in the high-cost account to improve resource allocation and optimize spending.
  d. Government must streamline access to somatic and specific mutation testing to reduce treatment delays and health care expenditure.
  e. Government must approve targeted therapies and companion molecular tests concurrently to promote early treatment application. Access to molecular diagnostic assays must be updated as new targeted agents are approved.
  f. The Dirección de Regulación de Beneficios, Costos y Tarifas del Aseguramiento en Salud, and the Ministry of Health must create specific molecular and genetic testing codes to include in PBS.
